# Greenhouse gas balance and mitigation of pasture-based dairy production systems in the Brazilian Atlantic Forest Biome

**DOI:** 10.3389/fvets.2022.958751

**Published:** 2022-09-23

**Authors:** Patrícia Perondi Anchão Oliveira, Alexandre Berndt, André de Faria Pedroso, Teresa Cristina Alves, Amanda Prudêncio Lemes, Bia Anchão Oliveira, José Ricardo Macedo Pezzopane, Paulo Henrique Mazza Rodrigues

**Affiliations:** ^1^Department of PD&I, Embrapa Southeast Livestock, São Carlos, Brazil; ^2^Veterinary Hospital, Brazil University, Fernandópolis, Brazil; ^3^School of Agriculture and Environment, Massey University, Auckland, New Zealand; ^4^Department of Animal Nutrition and Production, School of Veterinary Medicine and Animal Science of University of São Paulo, São Paulo, Brazil

**Keywords:** GHG emission intensity, carbon sequestration, enteric methane emission, eucalyptus, Nitrous oxide emission, mitigation GHG emissions

## Abstract

Brazilian cattle production is mostly carried out in pastures, and the need to mitigate the livestock's greenhouse gas (GHG) emissions and its environmental footprint has become an important requirement. The adoption of well-suited breeds and the intensification of pasture-based livestock production systems are alternatives to optimize the sector's land use. However, further research on tropical systems is necessary. The objective of this research was to evaluate the effect of Holstein (HO) and Jersey–Holstein (JE x HO) crossbred cows in different levels of pasture intensification (continuous grazing system with low stocking rate–CLS; irrigated rotational grazing system with high stocking rate–RHS), and the interaction between these two factors on GHG mitigation. Twenty-four HO and 24 JE x HO crossbred dairy cows were used to evaluate the effect of two grazing systems on milk production and composition, soil GHG emissions, methane (CH_4_) emission, and soil carbon accumulation (0–100 cm). These variables were used to calculate carbon balance (CB), GHG emission intensity, the number of trees required to mitigate GHG emission, and the land-saving effect. The number of trees necessary to mitigate GHG emission was calculated, considering the C balance within the farm gate. The mitigation of GHG emissions comes from the annual growth rate and accumulation of C in eucalyptus trees' trunks. The CB of all systems and genotypes presented a deficit in carbon (C); there was no difference for genotypes, but RHS was more deficient than CLS (-4.99 to CLS and −28.72 to RHS ton CO_2e._.ha^−1^.year^−1^). The deficit of C on GHG emission intensity was similar between genotypes and higher for RHS (−0.480 to RHS and −0.299 to CLS kg CO_2e._.kg FCPCmilk^−1^). Lower GHG removals (0.14 to CLS higher than 0.02 to RHS kg CO_2e._.kg FCPCmilk^−1^) had the greatest influence on the GHG emission intensity of milk production. The deficit number of trees to abatement emissions was higher to HO (−46.06 to HO and −38.37 trees/cow to JE x HO) and to RHS (−51.9 to RHS and −33.05 trees/cow to CLS). However, when the results are expressed per ton of FCPCmilk, there was a difference only between pasture management, requiring −6.34 tree. ton FCPCmilk^−1^ for the RHS and −3.99 tree. ton FCPCmilk^−1^ for the CLS system. The intensification of pastures resulted in higher milk production and land-saving effect of 2.7 ha. Due to the reservation of the pasture-based dairy systems in increasing soil C sequestration to offset the GHG emissions, especially enteric CH_4_, planting trees can be used as a mitigation strategy. Also, the land-save effect of intensification can contribute to the issue, since the area spared through the intensification in pasture management becomes available for reforestation with commercial trees.

## Introduction

Currently, Brazil occupies the third place in the world ranking of milk production, with more than 35 billion liters per year with dairy properties distributed in 98% of Brazilian municipalities (1). The majority of these farms are small and medium-sized; nevertheless, the sector employs close to four million people (2). More than 60% of the Brazilian milk production is concentrated in the Atlantic Forest Biome in the South and Southeast regions, with a herd of 8.05 million dairy animals and 24 billion liters per year. Brazil has 159.5 million ha of pastures, and of these, 42.2 million ha are in the Atlantic Forest Biome (1). Despite its scale of production, the trade balance of the national dairy sector had a deficit of US$ 378,840,000 in 2021 according to the Brazilian Foreign Trade Secretary (SECEX) (3). This discrepant scenario of systematic low efficiency can be explained by the production model adopted in the farms. Grasses are the main source of feed for dairy cattle, within pasture-based systems, which are often managed below their potential stocking rate, with a national average stocking rate of 1 cow.ha^−1^. In addition, the individual animal production is also low, 2,192 L. year^−1^ per cow. In the Southeast, for instance, the average milk production per cow is 2,580 L. year^−1^, and in the South, it is 3,618 L. year^−1^ (1).

While being challenged to meet the domestic demand for milk, within the past two decades the dairy sector has also been asked to satisfy the consumer demands for quality. Overall, consumers are more aware of issues related to value-added milk, product certifications (4), CB and the environmental footprint of milk and derivates (5, 6), and animal welfare regulations (7).

Among these requirements, the concern with climate changes stands out, considering that the agricultural sector is responsible for 33.6% of Brazilian GHG emissions, of which 19% are originated from enteric fermentation (8). The bovine herd contributes with 97% of enteric fermentation emissions, with 86% coming from the beef herd and 11% from dairy cattle (8). Subsequently to the ratification of several international agreements to combat the climate crisis by the Brazilian government, a strategic national plan was established in 2009, the ABC Plan, specifically “Mitigation and Adaptation to Climate Change for the Consolidation of a Low-Carbon Economy in Agriculture,” in accordance with the National Climate Change Policy as part of Brazil's action to mitigate GHG emissions and prevent global warming (9), currently called ABC+ Plan (Plan for adaptation and low C emission in agriculture 2020-2030) (10). Furthermore, Brazil joined the Global Methane Pledge, (11), which has as its main objective “to take voluntary actions to contribute to a collective effort to reduce global CH_4_ emissions by at least 30% of 2020s levels by 2030, which could eliminate over 0.2°C of global warming by 2050.”

Strategies to reduce greenhouse gas emissions, such as changes in the management of pasture-based dairy systems through the intensification of the forage utilization, and use of more specialized animal breeds and crossbreeds, have been reported also as effective CH_4_ mitigation strategies (12–16). Contrary to what was first believed, none of these approaches compromised animal performance, but presented an advantage when compared to the traditional Brazilian dairy systems; they became C sinks, by increasing the C sequestration of pasture-based systems (17–20). These actions (improve soil fertility and pasture management, animal breeding, dietary, and rumen manipulation) can positively contribute to the CB of dairy farms and diminish the necessity of outside actions (compensatory actions such as planting trees and purchasing C credits) to compensate and possibly offset their emissions.

Sustainable intensification of livestock production systems might become a key technology for the mitigation of climate changes. It is possible to infer that combining suitable animal genetics with functional pastoral systems is fundamental to improve dairy production and to guarantee the sustainability of the dairy sector. The results of long-term experiments in this area are scarce for tropical and sub-tropical regions.

Aiming to contribute to the improvement and sustainability of dairy production systems, this study evaluated the influence of two dairy cow genotypes and two levels of intensification in tropical pasture-based systems on milk yield, composition, soil GHG emissions, CH_4_ emission, and soil C accumulation rate in the depth of 0–100 cm (CAR), CB, GHG emission intensity, the numbers of trees necessary to compensate the GHG emissions, and land-saving effect of different dairy production systems.

The hypothesis of this study was that the use of crossbreed cows and intensified pastures contribute to the mitigation of GHG emissions and result in the land-saving effect.

## Materials and methods

### Site description

The experiment was carried out at Embrapa's Southeast Livestock Research Center, São Carlos, São Paulo, Brazil (22°1' S, 47°53' W; 853 m above sea level), during two periods: May 2012 to April 2013 and May 2013 to January 2014. The local climate is Cwa, according to Köppen's climate classification, with yearly average rainfall and temperature of 1,360 mm and 20 °C, respectively (21). The experimental location is characterized as Brazilian Atlantic Forest Biome (22). The soil profile is classified as dystrophic Red-yellow Latosol—Oxisol, according to the Food and Agriculture Organization of the United Nations (FAO) classification (Hapludox, after United States—US Soil taxonomy) with a sandy loam texture (23).

### Experimental design and treatments

The experiment used a randomized complete block design, with a 2 x 2 factorial arrangement of the treatments, defined as two cattle genotypes: Holstein (HO) and Jersey–Holstein (JE x HO), and two grazing management systems: extensive (CLS)—continuous grazing system with low stocking rate; intensive (RHS)—rotational grazing system, irrigated pasture with high stocking rate. The Native Forest (FOR)—Atlantic Forest (seasonal semi-deciduous forest), was used as a reference area to calculate C stocks. Each treatment had two area replications and two periods.

### Pastoral system history and management

The original forest biome (Atlantic Forest) was converted into *Brachiaria* spp. and *Cynodon nlemfuensis* Vanderyst pastures in 1984. The ranges were continuously grazed within seasons and throughout the years with adjustments in the animal stocking rate.

This original grazing system was adopted as the CLS pasture treatment and comprised of two paddocks of 3.0 ha each (area replication). Through the course of the experiment, the average levels of phosphorus (P) and potassium (K) in soil were 26.5 mg P dm^−3^ and 3.1 % K in soil CEC (cation exchange capacity), with no lime and fertilizer application.

After 10 years of continuous management (1984–1994), part of the grazing system area was converted into the intensively managed system. The RHS pasture was cultivated with *Panicum maximum* Jacq cv. Tanzânia and overseeded annually with *Avena byzantina* cv. São Carlos and *Lolium multiflorum* Lam. cv. BRS Ponteio in the autumn and irrigated. The irrigation criteria were according to Rassini (24), using the climatological water balance method. The RHS system consisted of two experimental units of 1.6 ha (replication area) that were divided into 27 paddocks (500 m^2^), subjected to rotational grazing, with 1 day of occupation and 26 days of resting.

The lime application in RHS systems consisted of 2.2 ton ha^−1^ of dolomitic limestone in the first year and one fertilizer application of 175 kg ha^−1^ year^−1^superphosphate in the second year to achieve 20 mg P dm^−3^. Fertilization with nitrogen (N) and K was made at the rate of 456 kg ha^−1^ year^−1^ and fertilization with sulfur (S) was made at the rate of 136.8 kg ha^−1^ year^−1^ (six applications of 40 kg N and K ha^−1^ in the rainy season and six applications of 36 kg N and K ha^−1^ in the dry season, using the 20-05-20 + 6% S formula). The N, K, and S fertilizer was made in each paddock at the first day after grazing.

Both grazing systems had their stocking rate adjusted according to Mott et al. (25). The “put and take” method adjusts the stocking rate periodically due to changes in the forage supply, aiming to keep the grazing pressure as close to the carrying capacity as possible throughout the experiment (26, 27). The forage availability was assessed as the grasses' stubble height (35 cm for pastures in RHS and 15 cm for pastures in CLS), according to Costa and Queiroz (28).

Cows were kept on pasture and received a concentrate supplement at a rate of 1:3 (kg of concentrate: kg of milk produced) and offered individually twice a day before milking. The concentrate was composed of 84.36% corn grain, 10% soybean meal, 3% vitamins and minerals mixture, 1% urea, 1.6% sodium bicarbonate, and 0.04% Rumensin (100 g kg^−1^ of sodium monensin). The concentrate was formulated according to the NRC (29) to have 16.3% crude protein (CP), 11.6% neutral detergent fiber (NDF), 5.5% acid detergent fiber (ADF), 3.9% ether extract, 62.8% starch, 67.8% non-fibrous carbohydrate, 6.1% ash, 0.48% Ca, 0.72% P, 82.3% total digestible nutrients (TDN), and 1.92 Mcal kg^−1^ of net energy for lactation. For the nutritive value composition, the forage samples were composed by season of the year, calculating the proportion of forage production in each season in relation to the annual forage production. Nutrient composition of forage (Table 1) was performed according to AOAC (30).

**Table 1:** Bromatological composition of the pastures (DM basis).

	**Pasture**	
**Item**	**Extensive**	**Intensive irrigated**
**Crude Protein** (%)	13.5	16.6
**Acid detergent fiber** (%)	35.1	34.7
**Neutral detergent fiber** (%)	68.6	65.7
**Lignin** (%)	6.8	6.2
***In vitro*** **dry matter digestibility** (%)	65.4	64.9
**Mineral matter** (%)	9.1	10.4

### Livestock management

The cows were managed according to the Institutional Animal Care and Use Committee Guidelines of Brazilian Agricultural Research Corporation—EMBRAPA, Brazil (PRT n. 5/2016). Twenty-four cows were used in each evaluation period (2012–2013 and 2013–2014), totaling 48 cows in the total experimental period (24 HO and 24 JE x HO crossbreed).

Cows were selected from an experimental herd, previously prepared for the experiment. All cows, inside of each genotype group, were uniform in age, live weight, stage of lactation, and milk yield, with cows in both groups initially at approximately 90 days of lactation. In the beginning of the experiment, the initial body weights (BW) were 609 (SEM = 10.3) and 548 (SEM = 13.6) kg, lactation numbers were 2.9 and 2.4, and days in milk were 60 and 100 days, for HO and JE x HO crossbreed, respectively. The cows were part of a breeding program, where JE x HO crossbreeds were produced from insemination of purebred HO cows (all belonging to the same herd) with Jersey bull semen. Holstein cows were originated from the same herd but using HO bull semen (purebred animals).

The experimental period comprised two lactations (2012–2013 and 2013–2014). Evaluations started in autumn and were completed in summer. Cows were mechanically milked twice a day (6h00 and 16h30). Milk production was evaluated every 15 days (during two consecutive days) using Milk Meters (EZI TEST/TRU TEST GROUP, New Zealand). Milk was sampled for analysis once a month. Samples were taken in each of the two daily milking, directly from the milk meters into flasks containing bronopol, and a composite sample was sent for analysis to a laboratory at the University of São Paulo (Clínica do Leite—ESALQ/USP). Fat, crude protein, lactose, total solids (TS), and dry defatted extract were analyzed by Infra-Red PO ANA 001, according to TUT PC 005 (31). The 3.5% fat and crude protein-corrected milk (FCPCmilk) was calculated according to NRC (29).

Individual milk production, milk composition, and stocking rate (cows ha^−1^) data were used to calculate the productivity of milk and its components per year (kg ha^−1^ year^−1^). All cows (testers or not) were weighed monthly. The daily stocking rate (cows ha^−1^ d^−1^) was obtained by dividing the total weight of cows present in each replicate pasture by the average weight of the experimental cows (tester) of each genotype and by the area of the pasture.

### Soil sampling and laboratory analysis

At the beginning of the experimental period (2012), soil samples (0–20 and 20–40 cm) were randomly taken for the characterization of the soil profile of the CLS system and for liming and fertilization in the RHS system. Soil samples were at 0–5, 5–10, 10–20, 20–30, 30–40, 40–60, 60–80, and 80–100 cm depths in six trenches (three replications per area) of 1.2 x 1.2 x 1.2 m dimensions (20). They were randomly located about 100 and 150 m afar in each treatment (CLS, RHS, and FOR) and utilized for soil chemical–physical analyses using the protocol by Fernandes et al. (32). Composite soil samples were also taken at the same depths (from 0–5 to 80–100 cm) at twelve sampling points across each trench to evaluate soil textural structure, OM, and C, according to the protocol by Fernandes et al. (32).

Soil samples were sieved and weighed for the measuring of clay and sand. Silt was defined by subtracting sand and clay weight from the initial weight of the sample (10 g) (33). The simplified textural class triangle was used to determine soil texture (23). Soil samples were air-dried and sieved (2 mm). Sub-samples were sieved (60 mesh) using a roller-mill grinding process (34). An elemental analyzer was used to define C percentage by dry combustion. Soil C stocks (0–30 cm and 0–100 cm) were assessed using the protocol proposed by Fernandes et al. (32), based on Ellert and Bettany (35) and Sisti et al. (36), corrected based on the soil mass of a reference area, the forest in this experiment. The corrected C stock was estimated by Equation 1:


(1) $$\begin{array}{l} Cs=\overset{n-1}{\underset{i=1}{\sum }}Cti+\left[Mtn-\left(\overset{n}{\underset{i=1}{\sum }}Mti-\overset{n}{\underset{i=1}{\sum }}Msi\right)\right]*Ctn \end{array}$$

Cs = total C stock (Mg ha^−1^), corrected based on the soil mass of a reference area;

$\sum_{i=1}^{n-1}Cti$ = sum of C stocks from the first to the next-to-last deepest layer sampled in the treatment (Mg ha^−1^);

Mtn = soil mass of the deepest layer in the treatment (Mg ha^−1^);

$\sum_{i=1}^{n}Mti$ = sum of total soil mass in the treatment (Mg ha^−1^);

$\sum_{i=1}^{n}Msi$ = sum of total soil mass in the reference area (Mg ha^−1^);

Ctn = soil C content in the deepest layer (Mg Mg^−1^ of soil).

Prior to the correction using soil mass, C stock of each layer was calculated using Equation 2 (37):


(2) $$\begin{array}{l} Cst=\;\frac{\left(CO^{*}Ds^{*}e\right)}{10} \end{array}$$

Cst = C stock in a certain layer (Mg ha^−1^).

CO = total organic C content in the layer (g kg^−1^).

Ds = soil density in the layer (kg dm^−3^).

e = layer thickness (cm).

Dividing the difference between C stock in the pasture systems and the one in the native forest (reference) by the number of years passed since the implementation of each pasture system until the soil sampling date (2012) was estimated the annual C accumulation rates for 0–100 cm layers, according to previous studies (19, 32, 38).

Soil organic C was determined by dichromatometry (33). Organic matter (OM) was extracted in sulfochromic solution (strong acid medium produced with sodium dichromate and sulfuric acid). The concentration of reduced Chromium (Cr III) ions was determined by colorimetry in a spectrophotometer, using 650 mm wavelength. The percentage of OM was calculated by multiplying the content of organic C by 1.724, this factor being derived from the C content in humus (58%).

Using a volumetric ring (Kopecky's Rings), undisturbed soil cores at the different layers of each trench were collected and used to evaluate the soil density. Bulk density was determined by dividing the dry weight of soil (105 °C) of each soil core by the volume of soil in the volumetric ring (33).

### Ruminal methane

The CH_4_ evaluation was made with sulfur hexafluoride (SF_6_) gas tracer technique (39), and refined by Berndt et al. (40). This technique is established (41) and can be applied with confidence in studies evaluating treatment effects (42), mainly for grazing animals (McGinn et al., 2006). This technique applies a calibrated permeation capsule placed in the rumen. In the first year, the calibration was 190 SF_6_ gas of 2.396 ± 0.06 mg. day^−1^, and in the second year, it was 190 SF_6_ gas of 1.753 ± 0.19 mg. day^−1^. The gases expelled through the mouth and nostrils were aspirated by a capillar tube adapted to a halter and connected to a canister under vacuum (collector), which was fixed on the neck of the animal. The CH_4_ collections were performed for five consecutive days, with the evacuated sampling canisters being changed every 24 h.

The sampling canisters were sent for chromatographic analysis, next to collection phase. Their contents were diluted with pure N to quantity of SF_6_ and CH_4_ gases, using a “Greenhouse” GC-2014 gas chromatograph (Shimadzu), with a flame ionization detector (FID) and an electron capture detector (ECD), respectively. The concentrations of CH_4_ and SF_6_ found in the “blank readings” were discounted from the concentrations found in the evacuated sampling canisters. The periods of sampling collects were in spring (September 21 to December 20), summer (December 21 to March 20), autumn (March 21 to June 20), and winter (June 21 to September 20). Methane emissions from enteric fermentation (t CO_2e._.ha^−1^ per year) were estimated using the Equations 3, 4, and 5.

CH_4_ emissions from enteric fermentation (t CO_2e._.ha^−1^ per year) = (annual average of animal's number .ha^−1^^*^ annual average emission of individual animal) ^*^ Global Warming Potential (GWP) ^*^ 365 days.     (3)

Annual average of animals number . ha^−1^ = (stocking rate daily spring average + stocking rate daily summer average + stocking rate daily autumn average + stocking rate daily winter average)/4, considering “put and take” method.     (4)

Annual average emission (CH_4_) of an individual animal (t. animal^−1^) = (CH_4_ daily spring average + CH_4_ daily summer average + CH_4_ daily autumn average + CH_4_ daily winter average)/4.     (5)

### Nitrous oxide and methane fluxes from pasture

**Figure 1:**
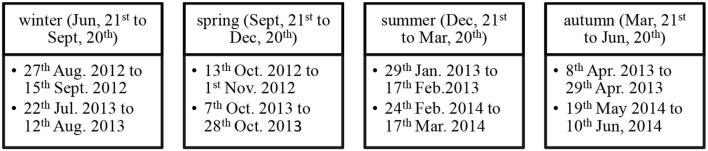
Flowchart of gas samples collect event basis.

The collected gas samples were accomplished on an event basis for 2 years, according to Figure 1.

PVC chambers installed in the experimental plots were used to collect gas samples (chamber technique), according to protocol suggested by Zanata et al. (43), based on Parkin et al. (44).

Three chambers were used per replicates (six per treatment). These were allocated randomly, not contemplating the possible presence of feces and urine from before. Instead, it was taken into consideration that in grazing systems with high stocking rates, feces and urine are distributed in the whole area making it difficult to detect places not contaminated by excreta (45). In every sampling period, samples were taken initially for five successive days and, subsequently, at two to three-day intervals until a total of 10-day samplings were finished in each season. This comprised 22 days of sampling per period. The first samplings occurred 24 h after fertilization, due to the use of N fertilizer in one of the treatments. Samples were collected between 8 and 10 a.m. Three individual samples were taken, after having fixed the chamber lids, at intervals of 0, 30, and 60 min. Whole samples were collected from 18 chambers (2 treatments and the forest x 6 chambers) for a total of 4,320 sampling events (18 chambers x 3 sampling times x 10 samplings x 4 seasons x 2 years). The analysis was realized in a Thermo Scientific™ TRACE™ 1310 GC with an automatic injector. The concentrations of CH_4_ and CO_2_ were defined with a flame ionization detector (FID) and the concentrations of N_2_O electron capture detector (ECD). External calibration was required to quantify the analytes. A calibration curve was generated with five different certified reference gas mixtures, each one containing SF_6_, CH_4_ + N_2_ balance with increasing quantities.

The gas increment for times (t0, t30, and t60) was calculated, considering the linear adjustment model and the molecular volume correction for the temperature inside the chamber (T) during sampling and using the formula (6) explained in the protocol suggested by Zanata et al. (43) and estimated the fluxes (F):


(6) $$\begin{array}{l} F=\left(\text{Δ}C\text{Δ}t^{-1}\right)x\left(MVm^{-1}\right)x\left(VA^{-1}\right), \end{array}$$

where ΔC Δt^−1^ represents the rate of change of the gas inside the chamber per unit of time (ppb/hour); M is the molecular weight (g); V and A are volume (L) and chamber area (m^2^), respectively; Vm is the molecular volume of the gas (L), corrected as a function of the temperature inside the chamber during sampling (1 mole of gas occupies 22.4 L under normal temperature and pressure conditions—CNTP), by multiplying 22.4 by (273 + T) / 273, with T being the average temperature inside the chamber in degrees Celsius.

Nitrous oxide and CH_4_ emissions from N fertilization and animal wastes (t CO_2e.._ha^−1^ per year) were calculated according to Equations 7, 8, and 9.

N_2_O emissions from N fertilization and animal wastes (t CO_2e.._ha^−1^ per year) = (annual average of emission N_2_O . ha^−1^^*^ GWP).     (7)

CH_4_ emissions from N fertilization and animal wastes (t CO_2e._.ha^−1^ per year) = (annual average of emission CH_4_ . ha^−1^^*^ GWP).     (8)

Annual average GHG emission from fertilization and animal wastes (t.ha^−1^ per year) = (daily spring average GHG emission^*^91.25 days) + (daily summer average GHG emission^*^91.25 days) + (daily autumn average GHG emission^*^91.25 days) + (daily winter average emission^*^91.25 days).     (9)

### Carbon balance and GHG emission intensities

Carbon balance was determined as the difference between the annual C accumulation rates of the grazing and the emissions of CO_2e._ derived from the dairy cattle production systems for 1 year (CH_4_ emissions from enteric fermentation, N_2_O and CH_4_ emissions from N fertilization and animal wastes), using AR6 – methodology (46) (GWP CH_4_=27.2, N_2_O=273) and the conversion factor of C to CO_2e._ = 3.67 (Equations 10 and 11).

CB (t of CO_2e._ /ha per year) = [(annual C accumulation rates 0-100 cm layers t.ha^−1^^*^ 3.67) – (annual emissions of CO_2e._ t.ha^−1^)].     (10)

Annual emissions of CO_2e._ (t of CO_2e._.ha^−1^ per year) = (CH_4_ emissions from enteric fermentation + N_2_O emissions from N fertilization and animal wastes + CH_4_ emissions from N fertilization and animal wastes), using AR6 (46) (GWP CH_4_=27.2, N_2_O=273) and the conversion factor of C to CO_2e._ = 3.67     (11)

The intensity of GHG emissions was calculated as the division between annual GHG emissions (t of CO_2e._.ha^−1^ per year) and the product output, FCPCmilk (kg.ha^−1^ per year).

The intensity of GHG removals was calculated as the division between annual C accumulation rates (t of CO_2e._.ha^−1^ per year) and the product output, FCCPmilk (kg.ha^−1^ per year).

The intensity of emission (CB emission intensity), considering the results of C balance, was calculated as the division between CB (t of CO_2e._.ha^−1^ per year) and the product output: stocking rate (cows.ha^−1^), milk (kg.ha^−1^ per year), FCPCmilk (kg.ha^−1^ per year), and ST (total solid; kg.ha^−1^ per year) of each treatment. The number of trees necessary to compensate the emissions of GHG from dairy cattle production systems was performed using these results.

### Annual C sequestration potential rate for Eucalyptus

Data from silvopastoral systems with *Eucalyptus* (333 trees. ha^−1^ during five first years and 166 trees. ha^−1^ over the next 3 years) in an additional experimental area located close to the area this study were collected in April 2016 and in April 2019. Forty trees (five years old) and 90 trees (8 years old), respectively, were utilized to define the wood volume and to acquire wood rings. Afterward, these samples were used to define biomass and C pools of the tree trunks. These findings were used to develop the Equations for the determination of stem volume and tree biomass. The Equations projected trunk volume at 215.2 m^3^ and trunk biomass at 98.9 t. ha^−1^ in the silvopastoral system. The diameter at the beginning and end of each segment and the segment mass were quantified. Afterward, a trunk sample (15 cm ring) was taken from each segment to establish the moisture content after oven drying at 60°C until constant weight. For these samples, density (ratio of dry mass to volume) and C content (by elemental Analyzer Perkin Elmer model CHNS 2400ii) were also defined (47).

Annual C sequestration potential rate for eucalyptus (CO_2e._.tree^−1^ per year) considered that 166 trees. ha^−1^ during 8 years and 167 during 5 years resulted in 98.9 t DM. ha^−1^ (215.2 m^3^ lumber) with 0.45 t C. t DM^−1^ that provided 75.6 kg CO_2e._. tree^−1^ per year, according to Equation 12.

Annual C sequestration rate (kg CO_2eq._.tree^−1^) = ((98.9 t DM . ha^−1^ x 0.45 t C. t DM^−1^)/((8 years^*^166 trees) + (5 years^*^167 trees)))^*^3.67 ^*^1000,     (12)

where DM = dry mass.

This result was utilized to estimate the number of trees required to mitigate the GHG emissions of grazing production systems.

### Land-saving effect and preservation of native forest

The “put and take” technique (25) using stocking rate adjustments was applied in all grazing systems, considering the forage availability of each paddock. The grasses' stubble height [30 cm for Panicum and 15 cm for Brachiaria, according to Costa (28)] was used as the method to adjust the pasture management. Annual average stocking rates were estimated for CLS and RHS pasture systems (20).

The land-saving effect of the intensified pasture system was calculated using equation 13, according to the methodology by Martha Jr. et al. (48):


(13) $$\begin{array}{l} Land-saving\;effect:\;\frac{\left(RHS\;SR-CLS\;SR\right)}{CLS\;SR} \end{array}$$

RHS SR = Intensive management stocking rate (animals. ha^−1^).

CLS SR = Extensive management stocking rate (animals. ha^−1^).

To assess the forest productivity components, the following C sinks were evaluated: (a) above-ground biomass, within necromass; (b) under-ground biomass (roots); and (c) litter. The experimental plots were designed according to the Brazilian National Forest Inventory (NFI) (20).

The diameter at breast height (DBH), tree height, and crown diameter (based on their projection onto the soil) were logged to estimate the above-ground biomass. Allometric models were registered to estimate the seasonal semi-deciduous forest biomass (20).

Samples of litter deposited in the floor were collected with a metal frame, and subsamples were utilized for C and DM analyses to measure and characterize the litter pool (20).

### Statistical analysis

Data were analyzed by the MIXED procedure of Statistical Analyses System (SAS) (49) after verifying for outliers and the residue normality by using the Shapiro–Wilk test (PROC UNIVARIATE, SAS Institute). For the analysis, among the 15 different covariance structures tested, the matrix that best fit to the data was chosen based on the lower corrected Akaike information criteria value (AICC) (50). The model included the effects of two types of pasture and two animals' genotypes and the interaction between pasture and genotypes (2 x 2). The Tukey test was used as the test to separate the means. The effect of periods and area replication was included in the model as random effect. The effects were considered significant at *p* ≤ 0.05.

## Results

### Milk productivity and carbon balance

**Figure 2:**
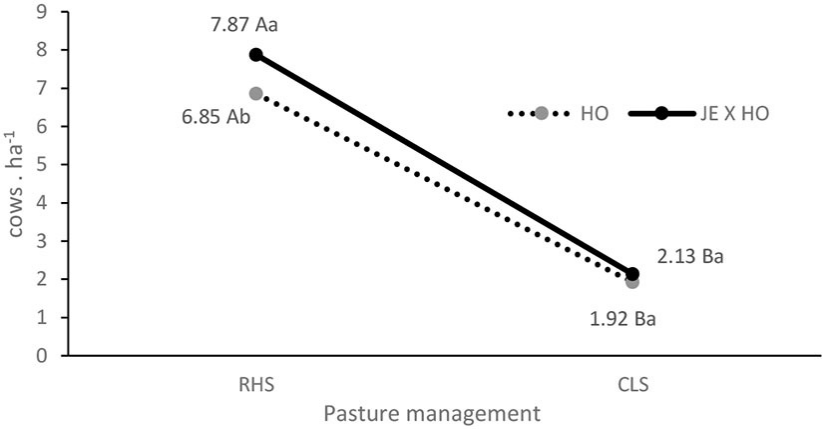
Interaction between two cow genotypes and two levels of intensification in grazing management systems for stocking rate. HO, Holstein cows; JE x HO, Jersey and Holstein cows crossbred; RHS, intensive—rotational grazing system, irrigated pasture with high stocking rate; CLS, extensive—continuous grazing system with low stocking rate. ^a − b;A − B^, means followed by different uppercase letters to pasture and lowercase to genotype are significantly different (*p* ≤ 0.05).

The animal stocking differed in the treatments with an interaction between pasture and genotype (P = 0.0178), such that in the CLS system, the stoking rate did not vary according to the animal genotypes, but in the RHS system the stocking rate was higher (7.87 cows.ha^−1^) for the JE x HO crossbreed than for the purebred HO cows (6.85 cows.ha^−1^) (Table 2, Figure 2). Despite this difference in the stocking rate, the other variables related to productivity (milk yield, corrected milk yield, and total solids) did not vary as a function of genotypes (Table 2).

**Table 2:** Stocking rate, milk yield, and its components for two cow genotypes and two levels of intensification in grazing systems.

**Item**	**Fixed effects**				**SEM^f^**	***P*** **-value**		
	**Genotype**		**Pasture management**			
	**HO^b^**	**JE x HO^c^**	**RHS^d^**	**CLS^e^**		**Pasture**	**Gen**.	Past*Gen
**Stocking rate** (cows.ha^−1^)	4.39	5.00	7.36	2.03	0.179	< 0.001	0.0017	0.0178
**Milk yield** (kg.ha^−1^.year^−1^)	40,095	41,611	63,867	17,839	1,914.3	<0.001	0.5936	0.9843
**FCPCmilk^a^ (kg.ha^−1^.year^−1^)**	36,915	38,231	58,918	16,229	1,790.2	<0.001	0.6136	0.9234
**TS** (kg.ha^−1^.year^−1^)	4,740.2	4,934.4	7,566.6	2,108.0	216.28	<0.001	0.5385	0.9674

a FCPCmilk, 3.5% fat and Crude Protein-Corrected Milk; TS, Total Solids of the Milk;

b HO, Holstein cows;

c JE x HO, Jersey and Holstein cows crossbred;

d RHS, intensive—rotational grazing system, irrigated pasture with high stocking rate;

e CLS, extensive—continuous grazing system with low stocking rate;

f SEM, standard error of the means.

The factor that most affected productivity was the intensification of pastures (*P* < 0.0001), which promoted an increase of more than 3.5 times in milk production, in FCPCmilk, and in total solids per hectare per year (Table 2). The CB, calculated as the difference between GHG emissions and GHG removals (Tables 3, 4), had a greater C deficit in RHS system compared with the CLS system (*P* < 0.0001), due to the higher GHG emissions (Table 3) and lower C removals (Table 4), resulting from a lower annual rate of C accumulation in RHS. Animal genotype did not influence GHG emissions (*P* = 0.3012) or the CB (*P* = 0.3207) of two production systems (Table 3).

**Table 3:** Greenhouse gas emissions and carbon balance for two cow genotypes and two levels of intensification in grazing management systems—Atlantic Forest as reference area to calculate carbon stocks.

**Item**	**Fixed Effects**				**SEM^g^**	***P*** **-value**		
	**Genotype**		**Pasture management**			
	**HO^c^**	**JE x HO^d^**	**RHS^e^**	**CLS^f^**		**Pasture**	**Gen**.	Past*Gen
**GHG emissions**^a^ (t CO_2e_.ha^−1^.year^−1^)	19.00	17.38	29.25	7.15	1.05	< 0.001	0.301	0.296
**C balance 0–100 cm^b^** (t CO_2e_.ha^−1.^year^−1^)	(–)17.44	(–)15.83	(–)28.27	(–)4.99	2.86	<0.001	0.321	0.316

t, 1,000 kg;

a GHG emission, Emission of greenhouse gases from animal and soil–plant systems, N fertilizers, and animal wastes; GWP 27.2 to CH_4_ and 273 to N_2_O (GWP100 from AR6);

b C balance, Carbon Balance;

c HO, Holstein cows;

d JE x HO, Jersey and Holstein cows crossbred;

e RHS, intensive—rotational grazing system, irrigated pasture with high stocking rate;

f CLS, extensive—continuous grazing system with low stocking rate;

g SEM, standard error of the means; ns = non-significant (p > 0.05);

^a − b^, means followed by different letters within a line are significantly different (p ≤ 0.05).

**Table 3.1:** Contribution of enteric CH_4_ and GHGs from the soil–plant system in the composition of GHG emissions for the interaction of two levels of intensification in grazing management systems and two cow genotypes.

**Fixed effects**		**CH_4_ enteric**	**N_2_O soil, plant system^a^**	**CH_4_ soil, plant system^a^**	**CH_4_ enteric**
**Pasture management**	**Genotype**	**t CO**_**2e**_**..ha**^**−1**^ **per year**			**%**
**CLS^e^**	**HO^b^**	7.13	0.00499	0.00047	99.92
**CLS**	**JE x HO^c^**	7.15	0.00499	0.00047	99.92
**RHS^d^**	**HO**	30.84	0.03569	−0.00465	99.90
**RHS**	**JE x HO**	27.59	0.03569	−0.00465	99.89

t, 1,000 kg;

a GHG emission, emission of greenhouse gases from animal and soil–plant systems, N fertilizers, and animal wastes; GWP 27.2 to CH_4_ and 273 to N_2_O (GWP100 from AR6);

b HO, Holstein cows;

c JE x HO, Jersey and Holstein cows crossbred;

d RHS, intensive—rotational grazing system, irrigated pasture with high stocking rate;

e CLS, extensive—continuous grazing system with low stocking rate.

**Table 4:** Annual carbon accumulation rate in the soil and removals of greenhouse gases of two levels of intensification in grazing systems—Atlantic Forest as reference area to calculate carbon stocks.

**Item**	**Pasture management**		**Mean**
	**RHS^c^**	**CLS^d^**	
**CAR^a^** **0–100 cm** (t C.ha^−1^.year^−1^)	0.26	0.59	0.42
**GHG removals^b^** **0–100 cm** (t CO_2e_.ha^−1^.year^−1^)	0.97	2.15	1.56

t, 1,000 kg;

a CAR, annual carbon accumulation rate in the soil—Atlantic Forest as reference.

b GHG removals = removals of greenhouse gases due to the annual carbon accumulation in the soil;

c RHS, intensive—rotational grazing system, irrigated pasture with high stocking rate;

d CLS = extensive—continuous grazing system with low stocking rate.

The output comparison of both production systems showed that the pasture intensification enabled the land-saving phenomenon to occur and had a great contribution to the system. The stocking rate in CLS was 2.03 cows ha^−1^, while the RHS stocking rate was 7.36 cows ha^−1^. As a result, the intensified system had a land-saving effect of 2.64 ha. In other words, once it is possible to produce the same amount of milk in 1.0 ha of RHS as in 3.64 ha of CLS, the intensification adopted allows the reforestation of the remaining 2.64 ha in RHS (Table 2).

### Greenhouse gas emission intensity

The C deficit in CB emission intensity per cow (*P* = 0.0552) and GHG emission intensity per FCPCmilk (*P* = 0.0355) was greater for HO purebred cows than for JE x HO crossbreed cows, but this difference was diluted when CB emission intensity was expressed by milk production (*P* = 0.4695), FCPCmilk (*P* = 0.4149), or TS (*P* = 0.4056), resulting in, this case, no difference between the genotypes (Tables 5, 6). This can be explained by a higher GHG removal intensity for HO purebred cows due to the lower product output per hectare, causing the ratio between the CAR/product output to be higher (Tables 2, 6).

**Table 5:** Emission intensity of greenhouse gases (GHG) for two cow genotypes and two levels of intensification in grazing management systems considering the systems carbon balance.

**Item**	**Fixed effects**				**SEM^g^**	***P*** **-value**		
	**Genotype**		**Pasture management**			
	**HO^c^**	**JE x HO^d^**	**RHS^e^**	**CLS^f^**		**Pasture**	**Gen**.	Past*Gen
**CB^a^** **emission intensity** (t CO_2_e.cow^−1^)	(–)3.520	(–)2.901	(–)3.924	(–)2.498	0.8278	0.0005	0.0552	0.1997
**CB emission intensity** (kg CO_2e_.kg milk^−1^)	(–)0.377	(–)0.339	(–)0.443	(–)0.274	0.0921	0.0071	0.4695	0.7891
**CB emission intensity** (kg CO_2e_.kg FCPCmilk^−1^)^b^	(–)0.413	(–)0.367	(–)0.480	(–)0.299	0.1005	0.0081	0.4149	0.8326
**CB emission intensity** (kg CO_2e_.kg TS^−1^)	(–)3.200	(–)2.840	(–)3.733	(–)2.308	0.7767	0.0063	0.4056	0.8153

t, 1,000 kg;

a CB, carbon balance;

b FCPCmilk, 3.5% fat and crude protein-corrected milk; TS, total solids of the milk;

c HO, Holstein cows;

d JE x HO, Jersey and Holstein cows crossbred;

e RHS, intensive—rotational grazing system, irrigated pasture with high stocking rate;

f CLS, extensive—continuous grazing system with low stocking rate;

g SEM, standard error of the means; ns, non-significant (p > 0.05);

^a − b;A − B^, means followed by different uppercase letters within a line to genotype and lowercase to pasture are significantly different (p ≤ 0.05).

**Table 6:** Emissions and removal intensity of GHG per milk yield (FCPCmilk) for two cow genotypes and two levels of intensification in grazing systems.

**Item**	**Fixed effects**				**SEM^g^**	***P*** **-value**		
	**Genotype**		**Pasture management**			
	**HO^c^**	**JE x HO^d^**	**RHS^e^**	**CLS^f^**		**Pasture**	**Gen**.	Past*Gen
**GHG^a^** **emission intensity** (kg CO_2e_.kg FCPCmilk^−1^)^b^	(–)0.495	(–)0.444	(–)0.499	(–)0.440	0.015	0.0184	0.0355	0.7484
**CHG removals intensity** (kg CO_2e_.kg FCPCmilk^−1^)	0.082	0.0772	0.0196	0.140	0.098	0.0159	0.9030	0.9108

a GHG, greenhouse gases;

b FCPCmilk, 3.5% fat and crude protein-corrected milk; TS, total solids of the milk;

c HO, Holstein cows;

d JE x HO, Jersey and Holstein cows crossbred;

e RHS, intensive—rotational grazing system, irrigated pasture with high stocking rate;

f CLS, extensive—continuous grazing system with low stocking rate;

g SEM, standard error of the means; ns, non-significant (p > 0.05); ^a − b;A − B^, means followed by different uppercase letters within a line to genotype and lowercase to pasture are significantly different (p ≤ 0.05).

The type of pastoral milk production system affected all CB emission intensity variables (*P* = 0.0005 to cow, *P* = 0.0071 to milk production, *P* = 0.0081 to FCPCmilk, and *P* = 0.0063 to TS), with the RHS system showing a greater C deficit for the CB emission intensity than the CLS system (Table 5).

To understand what influenced the results of the CB emission intensity of the different pastoral systems, the GHG emission intensity and the GHG removal intensity of FCPCmilk were calculated (Table 6). Although both GHG removals (0.14 to CLS higher than 0.02 to RHS kg CO_2e._. kg^−1^ FCPCmilk) and GHG emissions (−0.49 to RHS higher than −0.44 to CLS kg CO_2e._. kg^−1^ FPCmilk) contributed to the results in the GHG emission intensity of milk production (P=0.0184 and *P* = 0.0159, respectively), the greatest influence was due to the lower removals of GHG from RHS.

### Mitigation strategy inserting trees in the dairy cattle grazing production systems

With the results of CB and emission intensity, the quantity of trees necessary to mitigate GHG emissions in dairy cattle production systems per cow and per ton of product output was calculated (Table 7). The RHS system required more trees to mitigate GHG emissions per cow (*P* = 0.0005) and per t FCPCmilk (*P* = 0.0081) than the CLS system (Table 7).

**Table 7:** Trees needed to mitigate GHG emissions for two cow genotypes and two levels of intensification in grazing systems considering the systems carbon balance.

**Item**	**Fixed effects**				**SEM^g^**	***P*** **-value**		
	**Genotype**		**Pasture management**			
	**HO^c^**	**JE × HO^d^**	**RHS^e^**	**CLS^f^**		**Pasture**	**Gen**.	Past*Gen
**CB^a^** **mitigation trees** (number.cow^−1^)	(–)46.56	(–)38.37	(–)51.90	(–)33.03	10.949	0.0005	0.0552	0.1997
**CB mitigation trees** (number.t FCPCmilk^−1^)^b^	(–)5.46	(–)4.85	(–)6.35	(–)3.97	1.329	0.0081	0.4148	0.8324

t, 1,000 kg;

a CB, carbon balance;

b FCPCmilk, 3.5% fat and crude protein-corrected milk; TS, total solids of the milk;

c HO, Holstein cows;

d JE × HO, Jersey and Holstein cows crossbred;

e RHS, intensive—rotational grazing system, irrigated pasture with high stocking rate;

f CLS, extensive—continuous grazing system with low stocking rate;

g SEM, standard error of the means; ns, non-significant (p > 0.05);

^a − b;A − B^, means followed by different uppercase letters within a line to genotype and lowercase to pasture are significantly different (p ≤ 0.05).

The HO purebred cows required more trees (deficit of 46.56) than JE x HO crossbreed cows (deficit of 38.37) to mitigate GHG emissions intensity (*P* = 0.0552), but this difference did not occur when analyzed per product output (CB mitigation tress – number.t FCPCmilk^−1^) (Table 7). It followed the same pattern of results observed for CB emission intensity (kg CO_2e._. kg FCPCmilk^−1^), in which GHG removals were diluted in lower milk production of HO purebred cows, favoring the C balance (Table 5).

## Discussion

The results obtained for annual milk production were higher than the Brazilian average for the south and southeast regions (1). In the case of the extensive system (CLS), the best result was due to the superior genetics of cows in the experimental herd in relation to cows from farms in these regions. In the case of the intensive systems (RHS), it was due to the superior pasture (fertilized tropical pasture irrigated and overseed with oats and ryegrass in the dry and cold season) and the superior genetics of cows. The Brazilian Atlantic Forest Biome is located mainly in the south, southeast, and northeast regions, but the main milk production area of this biome is concentrated in the south and southeast regions, where the average milk production per cow is 3,618 and 2,580 L. year^−1^, respectively (1). The annual averages in this experiment were 8,788 and 8,678 L.cow^−1^ in CLS and RHS, respectively.

Under environmental conditions similar to those observed in our study, Teixeira et al. (51) estimated an annual milk productivity of 19,000 kg. ha^−1^ for an intensively managed and irrigated pasture (*Cynodon spp*. Tifton 85) with a stocking rate of 4.6 AU ha^−1^ (AU = Animal Unit 450 kg live weight). Alvim et al. (52) observed annual milk productivity of 28,430 and 37,959 kg milk ha^−1^ for irrigated coast-cross (*Cynodon dactylon*) pasture systems in which the stocking rates were 4.5 and 5.1 cows ha^−1^ and average individual daily productions of 16.9 and 20.0 kg milk per cow. day^−1^ and concentrates were fed at the rate of 3.0 or 6.0 kg per cow. day^−1^, respectively. Considering the average genotypes in this trial, the higher milk productivity observed for the intensively managed pastures may be attributed to the association of two factors: the higher stocking rate obtained in the irrigated pastures (7.2 cows. ha^−1^) and the higher individual milk production presented by the cows (24.5 kg FCPCmilk per cow. day^−1^) associated, of course, with higher concentrate supplementation (8.1 kg per cow day^−1^). These same factors influenced the best results obtained for total solids.

According to Oliveira et al. (20), the RHS presented higher stocking rate (7.35 cows. ha^−1^) that resulted in 2.64 ha of land-saving effect; meanwhile, the CLS stocking rate was 2.02 cows. ha^−1^ (Table 2). This means that for each 3.64 ha of CLS, 1.0 ha of RHS could be adopted and 2.64 ha could be reforested. Within this area contribution, it is possible to preserve approximately 145 different native tree species, besides the maintenance of several Atlantic Forest fauna species. The vegetal biomass from this area was estimated in 220.5 Mg ha^−1^, in which the total biomass was multiplied by 0.475 factor, according to Magnussen et al. (53) to calculate the total C stock in this area (104.6 Mg ha^−1^).

The CB per area unit is relevant to identify sources and sinks of atmospheric CO_2_ in an effort to develop strategies to mitigate anthropogenic emissions of GHG per hectare. The intensity of emission by unit of the products generated in each system is also of great relevance because this provides a wider perspective of the production chain footprint, once the efficiency related to each product of different systems is considered. In this context, the best scenario is minimum GHG emission combined with maximum productivity, in other words, a lower GHG emission intensity.

The higher GHG emission in the RHS system was expected due to the significant increase in animal stocking rate (Table 3) and consequent increase in enteric CH_4_ emissions. The enteric CH_4_ had the greatest emission contribution, being above 99% share in the composition of all emissions measured in each treatment (Table 3.1). An increase in C sequestration was expected in the more intensified and irrigated pastures, which did not occur (Table 4), leading to a greater deficit of C.

Most of the experiment findings related to GHG emission intensity and CB corroborated with the results of recent research papers (5, 6, 12, 13, 54). Cunha et al. (5) did an inventory of GHG emission on two Brazilian farms from Southeast region, Minas Gerais State, with different characteristics: farm 1—intensive (using semi-confined animal full time), and farm 2—semi-intensive production system (using pasture exclusive and silvopastoral systems, during rainy season; and sugar cane and corn silage supplementary forage, during dry season). The two farms used concentrate supplementation. The emissions (enteric CH_4_, animal wastes, N fertilization, oil, and electrical use) from intensive and semi-intensive production systems were 3.21 and 3.18 t CO_2_e.animal^−1^.yr^−1^, respectively. In this experiment, the results were similar, RHS presented 3.97 t CO_2_e.cow^−1^.yr^−1^, and the CLS, more extensive system, presented 3.52 t CO_2_e.animal^−1^.yr^−1^ (Tables 2, 3).

In several experiments involving livestock, the majority of GHG emissions originated from CH_4_ enteric fermentation. Cunha et al. (5) reported 67.1 to 71.4% of GHG emission from enteric fermentation, and they were of similar proportions on both farms evaluated by them. Lovett et al. (12), while working with dairy cattle and grazing systems in Ireland, observed that regardless of location, enteric CH_4_ production was the greatest single source of on-farm GHG emissions. The location affected the relative source strength (58 to 63%). In this experiment, as the N fertilization and oil and electrical use were not accounted, the enteric fermentation contributed with more than 99% of GHG emission (Table 3.1).

The C deficit in CB emission intensity per cow and GHG emission intensity per FCPCmilk was greater for HO purebred cows than for JE x HO crossbreed cows, but this difference was diluted when CB emission intensity was expressed by milk production, FCPCmilk, or TS. Congio et al. (15), evaluating crossbreeding as strategies to reduce CH_4_ mitigation, in a meta-analysis, observed that increase in milk yield of F1 Holstein × Gyr was linked to a reduction in CH_4_ per milk yield, while this was not the case for purebred Holstein. Pedreira et al. (13) also observed that Holstein cows produced more CH_4_ (299.3 g day^−1^) than the crossbred (264.2 g day^−1^). It can be speculated that diets from Latin America and Caribbean dairy systems, usually with lower energy content than typical dairy confinement diets from the USA, for example, may have restricted the potential of Holstein cows under such conditions, and privileged crossbreeds and more locally adapted cows (15).

The type of pastoral milk production system affected all CB emission intensity variables, with the RHS system showing a greater C deficit for the CB emission intensity than the CLS system (Table 5). The values found in this experiment were lower than those of Famiglietti et al. (6), which evaluated the C footprint of Grana Padano PDO cheese. Three Italian farms differing in herd size, housing systems, milk yield (kg milk cow^−1^ day^−1^), manure management systems, home-grown crops, and type and amount of production inputs found different direct on-farm GHG emission intensities, ranging from 0.57 to 0.80 kg CO_2e_.kg FCPCmilk^−1^. The emission intensity was also lower than the direct on-farm observed for the intensive farm (0.782 kg CO_2e._. L milk) and semi-intensive farm (0.974 kg CO_2e._. L milk) in Minas Gerais, Southeast region of Brazil (5).

In this trial, the enteric CH_4_ was associated with improvement in the stocking rate of the pasture intensification (Tables 2–3.1), as the stocking rate increased, the enteric CH_4_ emission obviously increased as well. It was possible to observe a response pattern as a function of levels of intensification in dairy systems—more GHG emission to intensive systems and majority of CH_4_ emissions originated from enteric fermentation. Then, in the context of the GHG emissions of pasture-based dairy production systems, reducing the enteric emission of CH_4_ emissions per animal is very important to ensure the intensification of grazing systems and concomitantly reduce GHG emissions, but this action can be carried out to a certain extent, even more in systems that use tropical pastures, which have lower quality compared to temperate pastures. However, management actions to improve the quality of tropical pastures should be thoroughly studied. Congio et al. (14), in a study in the Southeast region of Brazil, reported that CH_4_ emission per kg of milk was reduced by 21% with grazing strategies modifying the sward structure and improving the nutritive value of the forage.

The amount of GHG emissions originated from enteric fermentation in livestock production systems justifies the recent concern of the scientific community related to strategies to mitigate the emission of enteric CH_4_. Meta-analysis by Congio et al. (15), for the Latin America and Caribbean, and Arndt et al. (16), using global data, selected the best technologies to reduce the emission of CH_4_ and, at the same time, maintain or increase the animal performance, divided in three main categories: animal breeding, dietary manipulation, and rumen manipulation. However, these technologies have the potential to reduce enteric CH_4_ only by 10 to 30%, which is still not sufficient to meet international goals to reduce GHG emissions (Global Methane Pledge). This led the researchers into a new challenge of finding actions to complementarily mitigate the GHG emissions of livestock production systems.

In this scenario, soil C sequestration is an option to be considered. Grasslands can act as a significant C sink with the implementation of improved management (55). Various studies observed that C sequestration can be increased by adequate management practices such as rotational grazing and the use of appropriate carrying capacity (17–20, 56).

Hammar et al. (54), in Sweden, used a life cycle perspective to assess the climate impact of beef production and a modeling to calculate the C sequestration in soil and the potential offset enteric CH_4_ emissions. It showed an average C sequestration rate of 0.2 t C ha^−1^ and yr^−1^, so C sequestration could potentially offset 15–22% of GHG emissions from beef cattle production (enteric fermentation, feed production, and manure management), depending on system boundaries and production intensity (54). In another meta-analysis by Conant et al. (55), the results from 115 studies containing over 300 data points were analyzed. It concluded that the management improvements contributing to C sequestration were fertilization (0.30 t C ha^−1^. yr^−1^), improved grazing management (0.35 t C ha^−1^. yr^−1^), conversion from cultivation to pasture (1.01 t C ha^−1^. yr^−1^), conversion from native vegetation to pasture (0.35 t C ha^−1^. yr^−1^), sowing of legumes (0.75 t C ha^−1^. yr^−1^), improved grasses species (3.04 t C ha^−1^. yr^−1^), earthworm introduction (2.35 t C ha^−1^. yr^−1^), and irrigation (0.11 t C ha^−1^. yr^−1^), with a mean of 0.54 t C ha^−1^. yr^−1^; the results were highly influenced by biome type and climate (55). However, Oliveira et al. (19) observed negative annual C accumulation rate to irrigated intensified beef cattle system (-0.81 t C ha^−1^. yr^−1^).

This trial used the annual C accumulation rate observed by Oliveira et al. (20), as 0.26 and 0.59 t C ha^−1^ per year^−1^ to RHS and CLS systems (depth 0–100 cm), respectively. Although the C sequestration results in RHS were not the highest, they were better than those reported by Conant et al. (55) and Oliveira et al. (19) to irrigated grazing systems. But even so, they were not enough to offset all GHG emissions from the evaluated production systems. In this trial, although both GHG removals and GHG emissions contributed to the results in the GHG emission intensity of milk production, the greatest influence was due to the lower removals of GHG from RHS, reinforcing the fact that annual C accumulation rate was still not enough to remove the aimed GHG emissions from the evaluated production systems (Tables 3–5).

Considering that (1) enteric CH_4_ emission is the one that contributes more to the intensity of GHG emissions; (2) two recent and important meta-analyses (15, 16) evaluating PB (increasing feeding level, decreasing grass maturity, and decreasing dietary forage-to-concentrate ratio) and ABS strategies (CH_4_ inhibitors, tanniferous forages, electron sinks, oils and fats, and oilseeds) found a maximum potential of 35% reduction in enteric CH_4_ emissions; 3. annual soil C accumulation rate was still not enough to offset all GHG emissions, more alternative approaches are necessary to mitigate GHG emissions of milk chain.

In addition, Arndt et al. (16) related that globally, only if 100% of farms adopt the most effective PB and ABS strategies, it is possible to avoid the increase of 1.5°C in the average global temperature by 2030 but not 2050, because mitigation effects are offset by projected increases in CH_4_ due to increasing milk and meat demand. Notably, by 2030 and 2050, low- and middle-income countries may not meet their contribution to the 1.5 °C target for this same reason, whereas high-income countries could meet their contributions due to only a minor projected increase in enteric CH_4_ emissions.

Brazil, as middle-income country and the third largest milk producer in the world, needs to provide other alternatives to offset GHG emissions. The introduction of trees in the dairy systems can be an alternative strategy. We calculated that 3.97 to 6.35 trees per t FCPCmilk^−1^ and 33.0 to 51.9 trees per cow (Table 7) are necessary to mitigate deficit on farm gate C balance (Table 3).

According to Oliveira et al. (19), who calculated the number of trees needed to compensate GHG emission of pasture-based beef cattle, considering the C balance within the farm gate, the trees must be growing while the animals are being raised and estimates have been annualized. When animals are slaughtered, they can be replaced, and new animals can benefit from emissions mitigation from the annual development rate and accumulation of C in the trunks of the eucalyptus trees (19). In a similar condition, the C being accumulated in trees can be used for pasture-based dairy systems.

The trees can be inserted in the farm as a separated tree plantation, or as integrated system (livestock and forest), according to Oliveira et al. (19). In integrated systems, the trees would bring in a new set of dynamics; they would have an effect on pasture production, herbage growth rates in response to N applied, soil moisture, animal performance, and animal comfort, among other typical aspects of integrated production systems (19).

In addition, after cutting the trees, when they are transformed into harvest wood products (HWP), they do not instantly emit all the C stored in their products but have a decay according to the type of wood products use (46), and this value is not being computed in most CBs for livestock that use trees on their farms. To better understand the use of trees to offset GHG emission, it would be important to study the CB until finding an equilibrium scenario between GHG emissions of grazing-based livestock and the sequestration of C from the growth of commercial forests in farms in addition to the products made with the wood from these forests after cutting the trees (using Guidelines for National Greenhouse Gas Inventories—HWP, IPCC (46). It is worth pointing out that with each cut of the commercial forest with exotic species on the farms (Eucalyptus and Pinus mainly), usually the area is renewed in the Southeastern region of Brazil, by long-term economic interests and as a way of diversifying income.

Although the CB and GHG emission intensity observed in this trial is better than other results reported in the literature, it is still necessary to adopt other complementary GHG mitigation strategies. Due to the reservation of the pasture-based dairy systems in increasing soil C sequestration to offset the GHG emissions, especially enteric CH_4_, planting trees can be used as a mitigation strategy. Also, the land-save effect of intensified areas can contribute to the issue, since the portion spared through the intensification in pasture management becomes available for reforestation with commercial trees.

## Data availability statement

The raw data supporting the conclusions of this article will be made available by the authors, without unduereservation.

## Ethics statement

The animal study was reviewed and approved by Project No. 05/2016 is in accordance with the precepts of Law No. 11,794, of 8 October 2008, Decree No. 6,899, of 15 July 2009, and the rules issued by the National Council for the Control of Animal Experimentation, and was approved by the Ethics Committee on the use of Animals of Embrapa.

## Author contributions

PO, AB, and AP contributed to conception and design of the study. PO, TA, and AL organized the database. PR and BO performed the statistical analysis. PO wrote the first draft of the manuscript. JP wrote sections of the manuscript. All authors contributed to manuscript revision, read, and approved the submitted version.

## Funding

The authors would like to thank EMBRAPA for financing Pecus network (01.10.06.0001.05.00), CNPq for the financial support to the project 562861/2010-6, FAPESP for the financial support to the project 2017/20084-5, and CAPES x EMBRAPA (15/2014) for the scholarship and financial support to the project.

## Conflict of interest

The authors declare that the research was conducted in the absence of any commercial or financial relationships that could be construed as a potential conflict of interest.

## Publisher's note

All claims expressed in this article are solely those of the authors and do not necessarily represent those of their affiliated organizations, or those of the publisher, the editors and the reviewers. Any product that may be evaluated in this article, or claim that may be made by its manufacturer, is not guaranteed or endorsed by the publisher.
